# DGK-α: A Checkpoint in Cancer-Mediated Immuno-Inhibition and Target for Immunotherapy

**DOI:** 10.3389/fcell.2017.00016

**Published:** 2017-03-03

**Authors:** Elfriede Noessner

**Affiliations:** Immunoanalytics Core Facility and Research Group Tissue Control of Immunocytes, Helmholtz Zentrum MünchenMünchen, Germany

**Keywords:** tumor-infiltrating lymphocytes, renal cell carcinoma, diacylglycerol kinase, immunotherapy, anergy, human tumor

## Abstract

Immunotherapy is moving to the forefront of cancer treatments owing to impressive durable responses achieved with checkpoint blockade antibodies and adoptive T-cell therapy. Still, improvements are necessary since, overall, only a small percentage of patients benefit from current therapies. Here, I summarize evidence that DGK-α may represent an immunological checkpoint suppressing the activity of cytotoxic immunocytes in the tumor microenvironment. DGK-inhibitors can restore the antitumor function of tumor-suppressed adaptive and innate cytotoxic immunocytes. The activity of DGK-inhibitors lays downstream of current checkpoint blockade antibodies. Thus, synergistic effects are expected from combination strategies. Moreover, DGK-inhibitors may permit a double-strike attack on tumor cells as DGK-inhibition may not only re-instate immunological tumor attack but also may harm tumor cells directly by interfering with oncogenic survival pathways. Together, DGK-inhibitors have very promising characteristics and may be beneficially included into the armamentarium of cancer immunotherapeutics.

## Immunotherapy, approaching forefront of cancer therapy, searching for collaboration

Immunotherapy is moving to the forefront of cancer therapies with an increasing assortment of approaches being evaluated and approved for clinical application (Callahan et al., 2016; Papaioannou et al., 2016). Recent data document measurable improvement in patient outcome and, in several cases, induction of durable responses even in patients with far advanced disease that proved refractory to available therapies. The new therapies indicate a paradigmatic shift in cancer therapy in that tumor cells are no longer direct therapeutic target, but instead, therapies are directed toward the cells of the immune system restoring their ability to recognize and destroy tumor cells.

The immune system is ideally equipped to fight cancer as its components continuously travel throughout the body providing surveillance; immune cells can be specifically activated against tumors, which express antigen and are often immunogenic; and they can protect against tumor relapse owing to their ability to acquire specific and long-lasting memory. Yet, tumors escape from immune surveillance due to immunoediting (Dunn et al., 2004) and the development of immune cell dysfunctions (Frey and Monu, 2006; Gajewski et al., 2006). The new cancer immunotherapies became possible through a deeper understanding of the regulatory mechanisms that govern an effective immune response and technological advances in T-cell cultivation, engineering and antigen identification.

**Figure 1:**
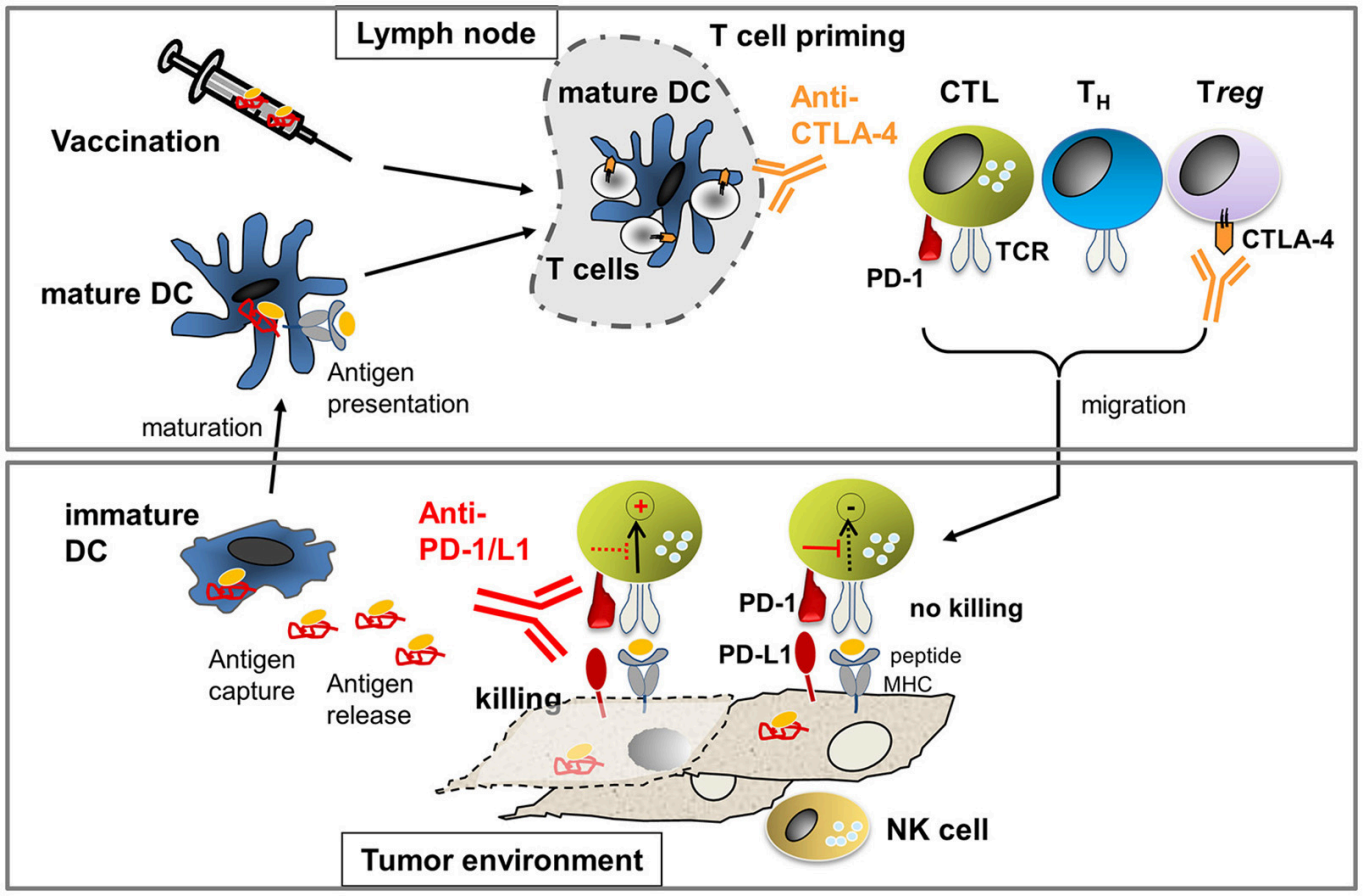
**Processes to activate T cell immunity against cancer: In the lymph node, T cell priming occurs through mature dendritic cells (DCs) presenting tumor-derived antigens**. The number of arising T helper (T_H_) cells and cytotoxic T cells (CTL) is limited through cytotoxic T-lymphocyte-associated antigen-4 (CTLA-4). Antibodies against CTLA-4 allow for activation of more T cells due to the amelioration of negative signals during T-cell priming. In addition, anti-CTLA-4 inhibits T*reg* which express high levels of CTLA-4 constitutively. Activated T cells migrate into the tumor milieu where they engage with tumor cells expressing peptide-MHC that can be recognized by the T cell receptor (TCR). TCR-pMHC interaction will activate tumor cell killing processes unless suppression occurs through concomitant PD-1/PD-L1 interaction. Killing of tumor cells can occur if the negative signaling is blocked through anti-PD-1 or anti-PD-L1 antibodies. NK cells can recognize tumor cells that express low or no MHC and, thus, cooperate with CTL to prevent tumor escape. If tumor cell killing occurs, antigen is released which can be taken up by immature DCs. Immature DCs can mature to mature DCs which then present antigen to T cells in the lymph node, leading to the generation of new tumor-reactive T cells. If the natural process of antigen presentation does not occur (efficiently), therapeutic vaccination using *ex vivo* generated antigen-loaded DCs or peptides may be applied.

The most advanced immunotherapeutic protocols to date include (Figure 1): (i) adoptive T-cell therapies, using *ex-vivo*-expanded autologous T cells isolated from tumor tissue (TILs) or autologous T cells engineered with therapeutic T-cell receptors (TCRs) or chimeric antigen receptors (CARs) recognizing tumor-expressed antigens; (ii) vaccination using tumor antigens or tumor antigen-presenting dendritic cells to stimulate the patient's immune system to generate tumor-reactive T cells *in situ*; and (iii) antibody-based therapies blocking immune checkpoints that would naturally elicit negative signals that hold back T cells to prevent autoimmune attack (Pardoll, 2012; Sharma and Allison, 2015).

While vaccination has yet to yield measurable clinical response (van der Burg et al., 2016), high and often long-lasting response rates are achieved with adoptive TIL therapy (Rosenberg and Restifo, 2015) and CD19-directed CAR-T cell therapy (Fesnak et al., 2016; Park et al., 2016). Yet, despite its high promises, adoptive T-cell therapy still faces significant hurdles to become one of the mainstay cancer therapies: TIL therapy is limited to tumor entities from which sufficient TILs can be procured [mainly melanoma and renal cell cancer (RCC)] and TCR- or CAR-T-cell therapy requires the knowledge of tumor-specific antigens to which T cells can be safely directed without harming vital organs. Currently, CAR-therapy is restricted to leukemia and lymphoma that express CD19 as targetable antigens. Treatment of solid tumors is explored, such as glioblastoma expressing a mutant form of the epidermal growth factor (EGFRvIII) or adenocarcinoma expressing cancer-associated glycoforms of mucin (Newick et al., 2016; Posey et al., 2016). Moreover, safety issues need to be resolved since serious adverse effects have been reported in TCR- and CAR-therapy trials (Gross and Eshhar, 2016).

Contrasting the currently limited application of adoptive T-cell therapy, immunotherapy with checkpoint blockade antibodies has achieved exciting results across a wide variety of cancer entities, not limited to commonly assumed immunogenic tumors such as melanoma or RCC, but also in lung cancer, bladder cancer or head and neck cancer. Three checkpoint blockade antibodies are currently in the clinic. One targets the cytotoxic T-lymphocyte-associated protein (CTLA)-4 (Postow et al., 2015; Sharma and Allison, 2015), which is an intrinsic negative regulator of T-cell activation during T-cell priming. The other two antibodies target the programmed death (PD) pathway through binding to the PD-1 protein or its ligand PD-L1. The PD-1/PD-L1 checkpoint is an extrinsic “off” signal that is operative in peripheral tissues turning off T-cell function to help control local inflammatory responses and maintain self-tolerance. Impressive durable responses have been observed using anti-CTLA-4 and anti-PD-1 resulting in their approval for the treatment of several cancers (Callahan et al., 2016). Yet, it has to be recognized that, overall, only a minority of patients experience substantial clinical benefit (around 15–40% depending on the tumor entity) (Sunshine and Taube, 2015; Ribas and Hu-Lieskovan, 2016). Improvements are necessary to unleash the full potential of immunotherapy and to potentially offer benefit to patients whose tumors are refractory to current therapies.

## Diacylglycerol kinase alpha: a checkpoint that negatively regulates T-cell function and curbs the activity of CD8-T and NK cells in the tumor microenvironment

T cells, in particular T_H_1/T_C_1-polarized lymphocytes, are important players in the antitumor response. Not only is their abundance associated with good prognosis in many tumor types (Fridman et al., 2012), they are also required for therapeutic response to checkpoint blockade therapy (Herbst et al., 2014; Tumeh et al., 2014). NK cells are innate cytotoxic lymphocytes appreciated for their ability to lyse virally infected cells as well as tumors. They play a complementary role to CD8-T cells as they recognize tumors which are resistant to T-cell killing due to downregulation or loss of MHC-class I molecules (refs. in Prinz et al., 2014). In some tumor types, such as RCC, they appear to play a prominent role as their number is predictive of good prognosis while that of CD8-T cells is not (Nakano et al., 2001; Eckl et al., 2012).

While the value of the natural immune infiltrate in tumors is appreciated, it has to be recognized that the natural immune response is not sufficient to control tumor progression in most cases. Various mechanisms are known that contribute to tumor immune escape ranging from ignorance to active suppression (Frey and Monu, 2006; Gajewski et al., 2006). One major hurdle is the inhibition of T-cell function in the tumor milieu. The suppressive quality of the tumor environment not only impacts the natural immune cell infiltrate but also curbs the efficacy of adoptive therapy, as even highly functional *in vitro* engineered CAR-T cell become unresponsive in solid tumor milieus (Janicki et al., 2008; Imai et al., 2009; Moon et al., 2014). Recognized mechanisms are T-cell exhaustion characterized by high expression of co-inhibitory receptors (PD-1, LAG-3, TIM-3) (Wherry et al., 2007; Pardoll, 2012), division arrest (Beyer et al., 2009), or effector phase inhibition due to disruption of TCR-signaling events (Frey and Monu, 2006).

We have analyzed the dysfunctional state of CD8-T and NK cells in human clear cell RCC (ccRCC) and observed a signature of anergy (Prinz et al., 2012, 2014). We found that TILs were non-responsive to stimulation, with much lower degranulation (measured by the appearance of CD107 protein on the cell surface), poor cytolytic activity (measured by chromium release assay) and low cytokine production compared to CD8-T cells and NK cells isolated from the non-tumor inflicted pole of the kidney (NILs) or from peripheral blood (PBLs). Mechanistically, no differences were seen activating proximal signaling molecules (Lck, ZAP70 or PLCγ) between TILs and NILs or PBLs; however, deficits in activating distal signaling molecules were evident. Identified key features included high levels of diacylglycerol kinase-α (DGK-α), low basal phosphorylation of the extracellular signal-regulated kinase (ERK) as well as reduced stimulation-induced phosphorylation of ERK, c-Jun N-terminal kinase (JNK) and AKT/protein kinase B. These features were caused by the tumor microenvironment as they were not observed in CD8-NILs or NK-NILs, and these lymphocytes were functionally active. The signature was similar to that previously described for anergic CD4^−^T cells (Macian et al., 2002; Zheng et al., 2008).

DGKs are appreciated as physiologic regulator of T- and NK-cell development, differentiation and function, through their activity to regulate the levels of DAG and phosphatidic acid (PA), which are important second messengers in the TCR-signaling cascade. The rasGRP/ERK pathway, activated by DAG, is crucial for MTOC-polarization, the delivery of lytic granules to the immunologic synapse (Quann et al., 2009) and the subsequent lytic attack on target cells. Cytotoxicity and production of IFN-γ, controlled among others by the ERK-pathway, are the most important effector activities required for tumor rejection. Thus, control of the ERK-pathway ultimately determines a T- and NK-cell's antitumor activity.

**Figure 2:**
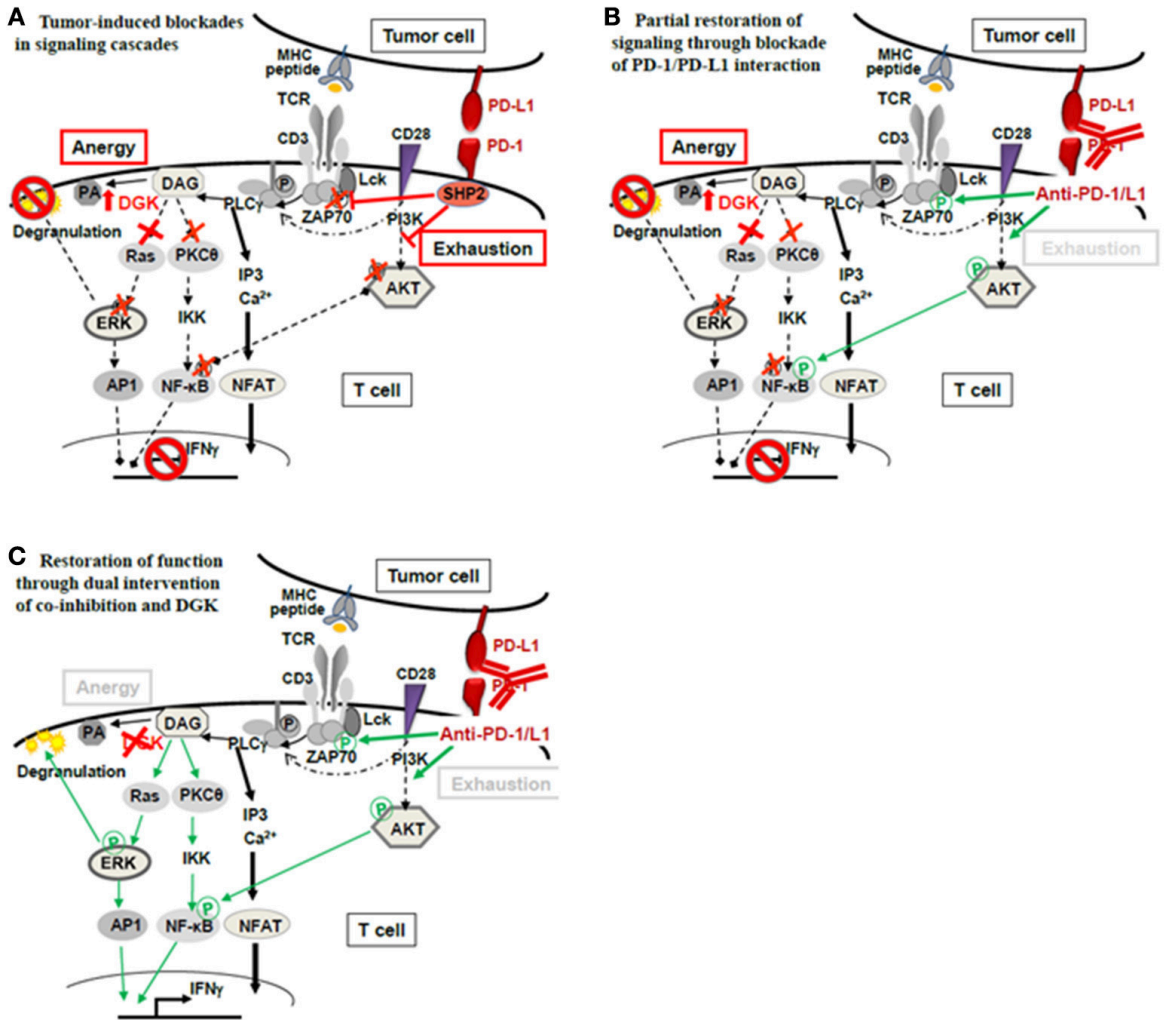
**Theoretical concept of combined application of checkpoint blockade therapy and DGK-inhibition. (A)** Functional unresponsiveness of TILs in the tumor milieu may have different mutually non-exclusive causes: (i) Ligation of PD-1 on T cells by tumor expressed PD-L1 may cause recruitment of phosphatase SHP2 and subsequent dephosphorylation of the TCR proximal signal transmitter Lck as well as attenuation of AKT-signaling. Consequently, signals initiated by TCR-peptide/MHC recognition intended to activate T-cell effector function (degranulation leading to lysis of target cells as well as IFNγ) are interrupted. The signal interruption through PD-1/PD-L1 classically occurs as a consequence of T-cell exhaustion. (ii) Anergy is another mechanism of T-cell silencing. The underlying cause is upregulated diacylglycerol kinase (DGK), in T cells mainly DGK-α and DGK-ζ. DGKs metabolize diacylglycerol (DAG) to phosphatidic acid (PA) lowering DAG levels which are necessary to activate TCR distal signaling through Ras/ERK. The ERK pathway is critically import for the degranulation process that delivers lytic proteins into the target cell for target cell death. **(B)** De-blocking the exhaustion pathway through checkpoint antibodies (anti-PD-1/PD-L1) releases the proximal brake at the TCR-associated molecules (Lck- and AKT-phosphorylation); however, distal brakes through DGK may still be active (blocked ERK pathway and attenuated PKC- and NFκB-activation) preventing full activation of the T cell‘s antitumor functions (degranulation, IFNγ). **(C)** Combined treatment with checkpoint antibodies and DGK-inhibitor may be required to open the signaling cascade fully, allowing effector function.

In experimental systems, overexpression of DGK led to a general attenuation of TCR-signaling as a direct result of decreased RasGRP1/Ras/ERK-pathway activation. Moreover, it has been shown that DGK-α and DGK-ζ, the abundant isoforms in T cells, are necessary for establishment of anergy (Zhong et al., 2008; Merida et al., 2015; Chen et al., 2016). Together, these experimental findings support our interpretation that T cells and also NK cells in the human RCC environment are anergic, showing overexpression of DGK-α, blunted ERK signaling and unresponsiveness to stimulation. Observing an anergic signature in TILs of ccRCCs is not unexpected since ccRCC is an epithelial tumor and, thus, largely devoid of co-stimulatory ligands, representing the classical anergy-inducing environment. Still, we do not rule out that additional mechanisms such as exhaustion or tolerance mediated through surface expressed co-inhibitory molecules such as PD-1, also contribute to functional unresponsiveness of TILs (Figure 2A). Rather, the causes of functional unresponsiveness in the tumor milieu are likely multifactorial. This is exemplified in an adoptive transfer experiment using CAR-T cells (Moon et al., 2014): in the tumor microenvironment, CAR-T cells rapidly became hypofunctional with identified upregulation of intrinsic T-cell inhibitory enzymes (DGK-α, DGK-ζ, SHP-1) as well as expression of surface co-inhibitory receptors (PD-1, LAG-3, TIM-3, 2B4).

## Reversal of tumor-induced suppression and restoration of T- and NK-cell activity through DGK-inhibition

IL-2 is a well-known growth factor for T and NK cells and has a history in RCC immunotherapy, achieving tumor control in a subgroup of patients (McDermott, 2009). IL-2 is known to regulate DGK-α and to restore functional responsiveness of anergic CD4-T cells (Macian et al., 2002). We could show that IL-2 restored *in vivo*-repressed cytokine secretion and cytotoxicity of CD8-TILs and NK-TILs. In CD8-TILs, functional recovery occurred concomitantly with a decrease in DGK-α and an increase in basal and stimulation-induced phosphorylation of key signaling proteins (ERK, AKT). In NK-TILs, IL-2 also restored activity; here, no change in DGK-α protein was observed suggesting direct regulation of ERK-phosphorylation, which is in accordance to published literature (Kondadasula et al., 2008).

We used the commercial DGK-inhibitor R-59022 and were able to document restored degranulation of CD8-TILs and NK-TILs, and, concomitantly, stronger ERK-phosphorylation, thus linking DGK-α to suppressed ERK-phosphorylation and inhibited degranulation. Of note is that the level of degranulation of TILs in the presence of DGK-inhibition was not higher than that observed with NK-NILs or CD8-NILs indicating that DGK-inhibition can restore suppressed degranulation but does not augment degranulation beyond an NK or T cell's intrinsic response efficacy. This finding helps alleviate concerns about potentially unleashing undesirable autoimmunity through DGK-inhibition, which is an important issue when considering potential targeting of DGK in a clinical setting.

## DGK-inhibitors to collaborate for effective cancer immunotherapy

DGKs, expressed in T and NK cells, are attractive targets for immunotherapy considering their physiologic function in regulating strength and duration of signaling cascades important for T- and NK-cell function. Observing that DGK-α and DKG-ζ are exploited by cancer cells to suppress the activity of cytotoxic immunocytes in the tumor microenvironment encourages the idea that DGK-inhibitors might enrich the current cancer immunotherapy armamentarium. Indeed, in experimental settings, T- and NK-cell activity can be enhanced and anergy development can be prevented through deletion or inhibition of DGK-α or DGK-ζ (Riese et al., 2011, 2013, 2016; Martínez-Moreno et al., 2012; Yang et al., 2016). Our results with TILs from human RCC further suggest that DGK-inhibition may not only prevent development of unresponsiveness but may also be able to restore activity of suppressed immune cells. Importantly, DGK-α inhibition can restore the function of CD8-T cells and NK cells. NK cells can destroy tumor cells with low or no MHC-class I proteins that may develop as escape variants after successful T-cell therapy and can be the cause of treatment failure. Development of escape variants may well be prevented in therapeutic settings that activates and maintains NK-cell function conjointly to the activation of a T-cell response (Fruci et al., 2013).

The multifactorial nature of tumor-induced unresponsiveness necessitates the application of multiple means to fully unleash the power of immunotherapy. Anergy has to be recognized as part of the inactivation process and, DGKs as its mediators, should be considered as an additional checkpoint controlling T-cell and NK-cell function, in addition to the currently appreciated co-inhibitory checkpoints (PD-1, LAG-3, TIM-3, CTLA-4) (Figure 2A). The classical co-inhibition molecules interrupt the signaling cascade at proximal steps (Lck, ZAP70, PI3K/AKT), while anergy-associated blockades are located further downstream. Thus, when co-inhibition is therapeutically alleviated through anti-PD-1/PD-L1 or anti-CTLA-4, signaling will still be halted by paucity of DAG through high DGKs (Figure 2B). Thus, if T-cell non-responsiveness (also) involves high DGK, it is expected that releasing distal signaling blockades, i.e., through DGK-inhibition, is required in addition to checkpoint blockade therapy to fully reverse T-cell suppression (Figure 2C). Currently, the combined application of checkpoint antibodies and DGK-inhibition is a theoretical concept and awaits supportive data from experimental models.

DGK-inhibition may further improve immunotherapy considering that CAR-T cells lacking DGK-ζ were found to be resistant to the suppressive cytokine TGF-ß (Riese et al., 2013; Arumugam et al., 2015). The molecular basis for the cross-talk between the two signaling cascades remains to be resolved. One explanation could be a digital conversion of ERK-phosphorylation to function, whereby function is enabled if ERK-phosphorylation is above a certain threshold (outlined also by Prinz et al., 2012). Higher ERK-phosphorylation reached through DGK-inhibition may enable T cells to maintain phospho-ERK levels above the threshold required for function in the presence of other suppressive signals.

## Further considerations for the development of DGK-inhibition for immunotherapy

DGK-inhibition has promising feature for immunotherapy. T and NK cells express two isoforms, DGK-α and DGK-ζ, which both regulate effector-lymphocyte function through controlling DAG-abundance. Will it thus suffice to inhibit only one isoform to help T and NK cells maintain function in the tumor milieu, or is the inhibition of both required?

DGK-α and DGK-ζ activities are comprehensively discussed in recent reviews, and thus are only briefly touched here (Merida et al., 2015; Chen et al., 2016; Singh and Kambayashi, 2016). As specific inhibitors of DGK-ζ are not available, the issue which one of the isoforms or whether both should be preferably inhibited to support T and NK cells in the tumor microenvironment cannot be adequately addressed at the moment. Data generated with knock-out mice are of limited information concerning effects of DGKs in the effector phase of the immune response since disturbances in the development, in particular observed development of hyporesponsive NK cells and altered development of regulatory T cells (T*regs*), may obscure effects that DGK-inhibition might have when applied to the already developed immune system. Inhibition experiments need to be performed using immune cells from the tumor environment, since here the immune escape processes are manifested that are the target of immunotherapy. For clinical extrapolation, experiments need to utilize human immune cells as significant differences exist, previously discussed by us and others (Prinz et al., 2012; Moon et al., 2014). Anergy-inducing conditions might arise much more frequently in humans, since CD8-T cell effector differentiation causes CD28 loss in humans (but not in mouse) which deprives human CD8-T cells from receiving co-stimulation. This may moderate the extent to which DGK-α or DGK-ζ participates in the regulation of DAG-mediated pathways in human and mouse models.

Different structural designs of DGK-α and DKG-ζ and accordingly different modes of activation allow some speculation as to which isoform possibly contributes more to the regulation of DAG-mediated signals in a specific situation. DGK-α, containing a calcium-binding EF-hand motif, is activated through Ca^2+^ ions, in addition to Lck-mediated phosphorylation, while DKG-ζ, lacking the calcium-binding EF motif, is not responsive to calcium signals and is activated through protein kinase C (PKC)-mediated phosphorylation. In physiologic situation where TCR-activation occurs concomitantly with co-stimulation, DKG-ζ may play the dominant role. In situations, however, where the co-stimulatory pathway is not provided, DGK-α will be disproportionally activated through Ca-induced conformational changes and Lck-dependent phosphorylation. TCR-stimulation without co-stimulation commonly occurs during effector phase activation of CD8-T cells in epithelial tissue or carcinomas due to the paucity of CD28 on human CD8-T effector cells and the lack of co-stimulatory ligands in epithelial tissues. Evidence for this scenario is seen in TILs of RCC that failed to activate the AKT pathway after TCR-stimulation while Lck- and PLCγ-activation occurred normally. Thus, the necessary signals (Lck, Ca^2+^) for DGK-α-activation are provided, with ensuing depletion of DAG and attenuation of effector activity. Extending on this, one might speculate that DGK-α is more relevant isoform to be targeted in cancer immunotherapy.

However, DKG-ζ was not analyzed in TILs due to the lack of specific reagents. Results from adoptive transfer studies using human CAR-T cells showed that DGK-α and DKG-ζ were upregulated in hyporesponsive CAR-T cells recovered from the tumor milieu of human xenografts (Moon et al., 2014). Here again DGK-ζ specific inhibition was not performed; thus, it remains to be addressed to which extent DKG-ζ overexpression contributed to the hypofunctional state of the CAR-T cells.

Considering DGK-inhibition for cancer immunotherapy attention should also be given to possible effects on other immune cells in the tumor microenvironment and the cancer cells themselves. DGK-α and DGK-ζ have effects on macrophages, dendritic cells and T*reg*s (Singh and Kambayashi, 2016). In macrophages and dendritic cells, DKG-ζ deficiency was found to be associated with impaired secretion of inflammatory IL-12 and TNF and impaired Th1-responses. Both isoforms were reported to inhibit the suppressive activity of T*reg*s. Considering the required function of myeloid cells and T*reg*s in antitumor response, DGK-inhibition (independent of isoform) may not yield desirable outcome. However, it has to be noted that none of the experiments were performed with tumor-educated cell types, thus the outcome of DGK-inhibition on antitumor immunity of myeloid cells and T*regs* remains an open question.

Concerning effects on cancer cells, contrasting outcomes are reported for DGK-α and DKG-ζ in the regulation of the NFκB-pathway, with DGK-α providing activation and DGK-ζ being inhibitory under inflammatory conditions (Tsuchiya et al., 2015). In such scenario, DGK-α inhibition would be a preferable intervention. Along this line, another study reported suppression of oncogenic survival pathways through DGK-α inhibition causing tumor cell death *in vitro* and reducing tumor growth in mice (Dominguez et al., 2013).

Collectively, DGK-inhibition has promising feature for cancer immunotherapy on multiple levels, re-invigorating T and NK cells for tumor cell attack, possibly making them resistant to TGF-ß suppression, and also weakening tumor cells directly. As DGKs and co-inhibitory surface proteins (PD-1, CTLA-4) control different steps in the signaling cascade, it is expected that DGK-inhibition will combine beneficially with current checkpoint blockade therapies or other immunotherapies. Further development is needed in the field of specific DGK-inhibitors (Sakane et al., 2016) and side-by-side comparisons of DGK-α and DGK-ζ inhibition to delineate the specific contribution that each of the isoforms might have in the restoration or maintenance of immune cell function in the tumor environment.

## Ethics statement

The human material was collected as part of the surgical procedure and only left-over material was used for research. The patients gave informed consent that their material may be used for research. The material for research was given anonymously; therefore, the study is considered ethically unobjectionable.

## Author contributions

EN wrote the article and performed the literature search.

### Conflict of interest statement

The authors declare that the research was conducted in the absence of any commercial or financial relationships that could be construed as a potential conflict of interest.
